# Psychotherapie – zwischen Gesetz und Unmöglichkeit

**DOI:** 10.1007/s00729-023-00220-4

**Published:** 2023-05-09

**Authors:** Marie-Theres Haas, Anatol Möller

**Affiliations:** 1 Fakultät für Psychotherapiewissenschaft, Sigmund Freud PrivatUniversität, Freudplatz 1, 1020 Wien, Österreich; 2 Wien, Österreich

**Keywords:** Psychotherapie, Gesetz, Ausbildung, Psychoanalyse, Psychotherapiewissenschaft, Akademisierung, Psychotherapy Act, Education, Psychoanalysis, Psychotherapy Science, Academization

## Abstract

Die Psychotherapie ist eine Disziplin, die Menschen mit psychischen Leiden anhand ihrer zur Verfügung stehenden Methoden behandelt. Das 1991 in Kraft getretene Psychotherapiegesetz schafft seit rund drei Jahrzehnten für diesen Berufsstand den rechtlichen Rahmen. Seit einiger Zeit wird über eine Novellierung des Gesetzes diskutiert. Inhalt einer möglichen Gesetzesreform soll primär die Akademisierung der Psychotherapie, wie sie sich in Österreich bereits seit einigen Jahren an wenigen (Privat‑)Universitäten vollzieht bzw. in anderen europäischen Ländern wie in Deutschland seit 2019 in die Wege geleitet worden ist, sein. Jede Zeit verlangt aufgrund ihres dynamischen Geschehens Veränderung. Veränderung als eine kreative Dekonstruktion bestehender Strukturen und gleichzeitig als Schaffung eines Raums, der Neues in sich birgt. Bei all den berufspolitischen Überlegungen muss stets eine Ethik der Ausbildung und klinischen Praxis mitgedacht werden. Dieser Beitrag setzt an diesem Punkt an und gibt einen Kurzabriss über den Status quo des Psychotherapiegesetzes sowie die zur Debatte stehenden inhaltlichen Änderungen einer eventuellen Gesetzesnovellierung. Darüber hinaus werden mögliche Chancen sowie Schwierigkeiten, die eine vollständige Akademisierung des Fachs mit sich bringen könnte, diskutiert.

## Abstract (en)

Psychotherapy is a discipline that treats people with psychological problems, using the methods available to it. The Psychotherapy Act that entered into force in Austria back in 1991 has now been providing a legislative framework for this profession for almost three decades. For some time, there has been discussion about taking a fresh look at this legislation. Any such change to the form of this legislation should primarily address an improvement to the academic status of psychotherapy such as that being practiced in Austria for some years at a few (private) universities as well as that initiated in other European countries such as Germany since 2019. Every era demands change to reflect its own dynamic course of events. Change serves as a creative deconstruction of existing structures while at the same time creating a space for new things to occupy. As well as all the considerations relating to vocational policy, an ethical dimension needs to be considered in relation to education and in clinical practice. This article addresses precisely that point and briefly maps out the status quo of psychotherapy legislation and the changes being debated in relation to a possible redrafting of that legislation. Over and above that, this article discusses the potential opportunities and difficulties of creating a fully comprehensive academic status for this field.

Rund 32 Jahre ist es her, dass in Österreich das erste Psychotherapiegesetz (PthG), das den Beruf von Psychotherapeut_innen als auch die dazugehörige Ausbildung gesetzlich regelt, in Kraft getreten ist. Spätestens seit diesem Zeitpunkt leistet die Psychotherapie einen offiziellen und darüber hinaus „wesentlichen Beitrag zur Versorgung psychisch Leidender in Österreich“ (Löffler-Stastka und Hochgerner 2021, S. 57). Psychische Erkrankungen stellen mittlerweile das häufigste Krankheitsbild der Republik dar (ebd.). Es verwundert daher nicht, dass bereits verschiedene vergangene Regierungsprogramme die Neuregelung der psychotherapeutischen Ausbildung zum Inhalt hatten.

Die Diskussion um die Novellierung des Gesetzes, das dem Berufsstand der Psychotherapeut_innen den rechtlichen Rahmen schafft, scheint trotz oder gerade wegen der zahlreichen Regierungskrisen nie abgebrochen zu sein. Obwohl das Gesetz die Form, das Äußere, der Psychotherapie vorgibt, wirkt es auch auf die inhaltliche, intime Ebene des Fachs ein. Konkret geht es bei der Novellierung vor allem um die Frage nach einer möglichen Akademisierung der Psychotherapie. Eine Frage, mit der sich bereits Freud (1919j [1918]) im Zusammenhang mit der Psychoanalyse auseinandersetzte. Während sich ein Teil der Analytiker_innen der damaligen Zeit zum Ziel setzte, „die Anerkennung der Psychoanalyse durch die Universitäten und ihre Aufnahme in den medizinischen Lehrplan zu erreichen“, tendierte ein anderer Teil dazu, „außerhalb dieser Institutionen zu bleiben“ und „die pädagogische Bedeutung der Psychoanalyse nicht gegen ihre ärztliche zurücktreten“ zu lassen (Freud 1935a, S. 34). Die Frage nach der fachlichen Zugehörigkeit der Psychoanalyse ist mittlerweile obsolet geworden, nachdem sich unter dem Begriff der Psychotherapie in Österreich 23 verschiedene psychotherapeutische Schulen, die mittlerweile eine eigenständige wissenschaftliche Disziplin und jeweils ein eigenes Verfahren im Umgang mit psychischem Leid entwickelt haben, versammeln. Nicht nur ungeschriebene, sondern auch geschriebene Gesetze werden von Zeit zu Zeit infrage gestellt. Das ist wichtig für die Gesetze (Derrida 2010 [1998], S. 194) und die Leerstelle, die sie in ihrer Nachträglichkeit zu füllen versuchen (Schneider 1999, S. 55). Was geschieht, wenn ein Gesetz nicht mehr infrage gestellt wird oder werden darf, führt uns die Welt vor Augen. Etwas als endgültig anzunehmen und nicht mehr zu hinterfragen, birgt Gefahren. In diesem Sinne hat auch Balint (1953, S. 689) folgende Gedanken mit dem Berufsstand geteilt: „Wir könnten keinen größeren Fehler machen, als wenn wir unser gegenwärtiges Ausbildungssystem als endgültige Lösung unserer vielen Probleme ansehen wollten. Denn von einer wirklichen Lösung sind wir noch weit entfernt.“ Was für die Psychoanalyse gilt, ist für das gesamte Fach der Psychotherapie nicht minder von Bedeutung. *Unmögliche Berufe* wie der der Psychotherapeut_innen „sind in ihrer Ausübung vom Hin und Her der Übertragung gekennzeichnet. Sie fallen immer wieder aus der Gegenwart heraus, in der gearbeitet wird, arbeiten mit der Zukunft und rühren die Vergangenheit auf, verwirbeln die Zeiten im Moment“ (Pazzini 2022a, S. 158). Doch *wie* kann etwas Derartiges gelehrt werden (Bernfeld 1984 [1952], S. 439)? Die Autor_innen dieses Artikels sind von einem psychoanalytischen Hintergrund geprägt, wenngleich sie von einer wesentlichen Gemeinsamkeit aller anerkannten Psychotherapiemethoden ausgehen, die in der Dimension des Beziehungshaften liegt. Daher stellt der vorliegende Beitrag eine Verbindung zwischen dem Status quo des Psychotherapiegesetzes und der geplanten Novellierung her, bündelt mögliche Ängste vor dieser im Raum stehenden Veränderung und diskutiert das Potenzial einer vollständigen Akademisierung des Fachs.

## Der bisherige Weg

Bevor das Psychotherapiegesetz 1991 seine Rechtsverbindlichkeit erhielt, liefen Personen, die ohne ärztlichen Hintergrund psychotherapeutisch tätig waren, Gefahr, des Vorwurfs „des Verstoßes gegen ärzterechtliche Bestimmungen des Ärztevorbehaltes“ und des Verdachts auf „Kurpfuscherei“ belangt zu werden (Schmuck und Kierein 2020, S. 655). Der Fall Theodor Reik ist ein Beispiel aus dieser „gesetzlosen“ Entwicklungsphase der Psychotherapie (Freud 1926e, 1927a). Mit dem Gesetz von 1991 konnte die Psychotherapie auch von Menschen, die kein Medizinstudium absolviert hatten, praktiziert werden. Als freier Beruf konzipiert, unterliegt es Psychotherapeut_innen selbst, ihre Tätigkeit „persönlich und eigenverantwortlich“ auszuüben (Schmuck und Kierein 2020, S. 655). Die Ausbildung zu Psychotherapeut_innen ist zweistufig aufgebaut und setzt sich entsprechend aus einem allgemeinen Teil, dem psychotherapeutischen Propädeutikum, und einem besonderen Teil, dem psychotherapeutischen Fachspezifikum, zusammen (§ 2 PthG).

Zu den Voraussetzungen zur Absolvierung des Propädeutikums zählen unter anderem eine erfolgreich abgeschlossene Reifeprüfung im In- oder Ausland, die Ausbildung im Krankenpflegefach- bzw. medizinisch-technischen Dienst oder die Einholung eines gesonderten Gutachtens (§ 10 (1) PthG). Das Fachspezifikum dürfen alle Personen absolvieren, die das 24. Lebensjahr vollendet haben, das psychotherapeutische Propädeutikum erfolgreich absolviert haben und entweder andere Voraussetzungen wie ein entsprechendes Gutachten, eine Ausbildung bzw. ein Studium an einer Akademie für Sozialarbeit, an einer Pädagogischen Akademie, an einer Lehranstalt für Ehe- und Familienberatung bzw. ein Studium der Musiktherapie, der Medizin, der Pädagogik, der Philosophie, der Psychologie, der Publizistik- und Kommunikationswissenschaft, der Theologie oder ein Lehramtstudium bzw. ein ausländisches Äquivalent zu den genannten Fachrichtungen vorzuweisen haben (§ 10 (2) PthG). Wer beide Ausbildungsschritte erfolgreich absolviert und das 28. Lebensjahr vollendet hat, kann den Antrag auf die Eintragung in die Psychotherapeutenliste stellen und ist nach entsprechender Genehmigung zur Berufsausübung berechtigt (§ 11 PthG).

Die psychotherapeutische Ausbildung führen derzeit in Österreich 21 propädeutische und 40 fachspezifische Ausbildungseinrichtungen durch (BMSGPK 2023). Es sind 23 psychotherapeutische Methoden anerkannt, die in vier Gruppen eingeteilt werden und die jeweils „auf unterschiedlichem theoretischen, philosophisch-erkenntnistheoretischen und anthropologischen Hintergrund basieren“ (Löffler-Stastka et al. 2018, S. 227). Neben dem ursprünglichen außeruniversitären Ausbildungsweg haben sich in den letzten Jahren auch Kooperationen zwischen externen Ausbildungsinstituten und (Privat‑)Universitäten ergeben, die die fachspezifische Ausbildung zum Beispiel im Rahmen eines Universitätslehrgangs oder gar eines eigenen Studiums anbieten. Die Sigmund Freud PrivatUniversität nahm und nimmt hier eine gewisse Vorreiterrolle ein, nachdem sie mit dem Studium der Psychotherapiewissenschaft seit 2005 als erste Universität weltweit die psychotherapeutische Ausbildung im Zuge eines umfassenden Bachelor- und Masterstudiums, das die Kriterien des Bologna-Systems erfüllt, anbietet (Pritz 2020, S. 20; Stephenson 2020, S. 207; Fiegl und Sindelar 2020, S. 287). Im Laufe der Zeit folgten dieser Idee in Teilen auch andere Universitäten wie die Karl Landsteiner Privatuniversität in Krems, die Bertha von Suttner Privatuniversität in St. Pölten oder auch die International Psychoanalytic University in Berlin.

## Schwierigkeiten

Trotz der „Flexibilität und Durchlässigkeit des österreichischen Psychotherapiegesetzes“, durch das es „bis dato den Veränderungen des Bildungs- und Gesundheitssystems seit 1990 standhalten“ konnte (Schmuck und Kierein 2020, S. 665), wird in letzter Zeit vermehrt über eine Novellierung des Gesetzes gesprochen. Hierfür mag es mehrere Gründe geben. Zum einen ist die Studienlandschaft durch den Bologna-Reformprozess und die Transformation zahlreicher Berufsausbildungen in Hochschul- bzw. Universitätsstudiengänge stark erweitert und damit unübersichtlicher geworden (ebd.). Ausbildungen, die früher mit einem Diplom beendet wurden, werden heute mit einem Bachelor oder Master abgeschlossen (ebd., S. 664). Das führt zu zunehmenden Komplikationen hinsichtlich der im Psychotherapiegesetz formulierten Quellberufe als Zugangsvoraussetzung (ebd.). Das genannte Problem der akademischen Einordnung und Vergleichbarkeit mit anderen bereits akademisierten Berufsausbildungen betrifft jedoch auch zum anderen die Psychotherapie selbst. So wird oftmals betont, dass die Psychotherapie die wesentlichen Merkmale einer eigenen Disziplin erfülle und längst Anspruch darauf habe, als eine eigenständige humanwissenschaftliche Disziplin anerkannt zu werden (Fischer 2011). Der aktuell noch zweigleisige Weg hat zur Folge, dass es eine Diskrepanz zwischen praktisch und forschend tätigen Psychotherapeut_innen gibt (Rieken 2013, S. 290). Praktiker_innen nehmen „die Ergebnisse der empirischen Forschung in der Regel nicht wahr, weil sie diese für wirklichkeitsfern halten, während empirische Forscher die praktische und theoretische Arbeit der Therapeuten kritisieren, indem sie ihnen mangelnde Wissenschaftlichkeit vorwerfen“ (ebd.). Zudem sind aufgrund der zunehmenden Globalisierung und der damit einhergehenden Digitalisierung und Mobilität neue therapeutische Settings entstanden, deren rechtliche Regelung noch nicht vollends im Psychotherapiegesetz Eingang gefunden hat. Die COVID-19-Pandemie hat diese Tendenzen beschleunigt und weitere Fragen aufgeworfen, die in einer Gesetzesnovelle berücksichtigt werden müssten. Ein selten genanntes Argument für die Novellierung des Psychotherapiegesetzes sind die Schwierigkeiten bei der Versorgung von psychisch Leidenden in Österreich. Laut Löffler-Stastka (2022, Min. 4:30) erreichen Psychotherapeut_innen und Ärzt_innen für psychotherapeutische Medizin gemeinsam lediglich 3 % der Bevölkerung, wohingegen der tatsächliche Bedarf bei rund 25 % liege. Dieser Versorgungsmissstand hängt möglicherweise neben dem geringen Angebot an kassenfinanzierten Psychotherapieplätzen und dem Fehlen eines Gesamtvertrags (Löffler-Stastka und Hochgerner 2021, S. 58) auch mit dem derzeitigen Psychotherapiegesetz und dem darin vorgegebenen Ausbildungsweg zusammen.

## Was ist geplant?

Im Zuge einer Novellierung des Psychotherapiegesetzes könnte die Psychotherapie als eigenständiges Direktstudium entsprechend den Vorgaben des Bologna-Systems gestaltet werden. Die psychotherapeutische Ausbildung würde sich dann durch ein Grundstudium, den Bachelor, ein darauffolgendes Aufbaustudium, den Master, und gegebenenfalls eine Fachausbildung als dritten Schritt auszeichnen (Schmuck und Kierein 2020, S. 666). Abgesehen von der Ausbildungsreform müssten laut Schmuck und Kierein „noch weitere Inhalte Eingang in den Gesetzestext nehmen […], wie beispielsweise die Erweiterung, Strukturierung und Konkretisierung der Berufspflichten, die Erweiterung der Sanktionsmöglichkeiten von Berufspflichtverletzungen sowie die Änderung der Struktur und Aufgaben des Psychotherapiebeirates“ (ebd.). Abgesehen von der Formulierung deskriptiver Zugangsvoraussetzungen wie einer „akademischen Erstqualifikation aus dem Gesundheits‑, Bildungs- und Sozialbereich […] könnten auch psychotherapeutische Ambulanzen bzw. psychotherapeutische Hochschulambulanzen als Lehr- und Forschungsambulanzen zu Ausbildungs‑, Forschungs- und Versorgungszwecken dienen“ (ebd., S. 666). In Anbetracht der vollständigen Kostenübernahme der Psychotherapie seitens der Krankenkassen in Deutschland und der im aktuellen Regierungsprogramm definierten Ziele könnte die Diskussion um eine umfangreichere Kostenübernahme der Psychotherapie in Österreich „im Zusammenhang mit dem Reformprozess des österreichischen Psychotherapiegesetzes“ neu entfachen (ebd., S. 670).

Die Ängste und Bedenken, die hinsichtlich dieser mal mehr mal weniger zur Diskussion stehenden Gesetzesnovellierung innerhalb der Community kursieren, sind mannigfaltig. Neben der Angst, dass die bestehenden Ausbildungsvereine nicht in die Gesetzesnovellierung einbezogen werden bzw. manche durch die Gesetzesnovelle vielleicht sogar in ihrer Existenz bedroht sind, besteht die Furcht vor einer überstürzten Gesetzesänderung, einem Qualitätsverlust der psychotherapeutischen Ausbildung, einem Verlust des Eklektizismus, einer Standardisierung und Rationalisierung des Berufsbilds und damit einhergehend einer geringeren beruflichen Freiheit. Des Weiteren gibt es Bedenken gegenüber jüngeren und dadurch mit geringerer Lebenserfahrung ausgestatteten Psychotherapeut_innen, bezüglich nicht ausreichend vorhandener kostenloser Ausbildungsplätze und weiter steigender Kosten der psychotherapeutischen Ausbildung.

## Das kreative Potenzial einer vollständigen Akademisierung

Eine wesentliche Intention der Gesetzesnovellierung ist es, die Psychotherapie zu akademisieren. Das bedeutet, dass ein Teil des bisherigen Ausbildungssystems von Psychotherapeut_innen, wie wir es bislang aufgrund des bestehenden Gesetzes kennen und es praktisch angewandt wurde, verändert und in manchen Teilen sogar dekonstruiert wird. Denn bisherige außeruniversitäre Ausbildungsstätten stehen mit einer möglichen Gesetzesnovellierung, die eine ausschließliche Akademisierung der Psychotherapie ins Visier nimmt, auf dem Prüfstand, insofern keine Kooperationen mit Universitäten eingegangen werden können. Eine Akademisierung würde also in rein formaler Hinsicht einen Prozess in Gang setzen, der das bestehende System zumindest teilweise auflöst, sodass erst durch diese Dekonstruktion ein neues System, nämlich eine vollständige Akademisierung des Fachs, möglich werden würde. Ein Erneuerungsvorgang, der mit dem im Westen vorherrschenden Kapitalismus in Zusammenhang gebracht werden kann (vgl. dazu MEW Bd. 23, S. 485) und an anderer Stelle auch als Prozess einer *Creative Destruction* bezeichnet wird (Schumpeter 2003 [1943], S. 83). Doch wie können wir uns diese geplante Akademisierung vorstellen? Und vor allen Dingen: Wie könnte diese gelingen? Befindet sich doch das Gros der öffentlichen Universitäten in einer prekären Lage, nachdem sie vom Staat sparsam budgetiert werden, zunehmend auf Ökonomisierung ausgerichtet sind, einem oftmals *leeren Sprechen* hinsichtlich angekündigter Reformen unterliegen und in vielen Fällen der Produktivität anstatt der Kreativität gerecht werden müssen. In diesem Zusammenhang wird an manchen Stellen auch von einer *Krise* der Universität (Adorno 2010 [1963], S. 181) und dem Bildungssystem im Allgemeinen gesprochen (Nida-Rümelin 2014, S. 13). Doch zunächst zu der ersten Frage:

Eine Akademisierung der Psychotherapie bedeutet demnach eine ausschließlich universitäre Ausbildung der Psychotherapie. Psychotherapie könnte also mit einer solchen Gesetzesnovellierung zukünftig ausschließlich an Hochschulen, sprich Universitäten oder Fachhochschulen, gelehrt werden. Sowohl Universitäten als auch Fachhochschulen sind höhere Bildungseinrichtungen. Stätten, an denen etwas gebildet wird, also etwas Neues im Entstehen ist. Orte, an denen sich Dinge und auch *Un*dinge (Haas 2022) möglicherweise in Bewegung setzen können. Die Universität als ein Ort, der in seiner Namensetymologie aus dem Lateinischen *universitas(-atis)* stammt und in seiner Übersetzung als Ganzes oder Gesamtheit in die gegenwärtige Sprache übersetzt werden kann. Ein Ort, an dem der Mensch Wissen schafft. Wie schafft er das? Indem er forscht, argumentiert, begründet und das Erforschte in einem Wissenstransfer von der Theorie in die Praxis überträgt – in Form von beispielsweise Lehren oder Publizieren, jedenfalls einem Teilen des generierten Wissens mit der wissenschaftlichen Community, einem Teilen mit dem *Ganzen*. Obwohl in der alltäglichen Sprache oftmals von der Universität die Rede ist, sprich mit bestimmtem Artikel und im Singular, unterliegen die vielen Hochschulen keinem einheitlichen Wissenschaftsbegriff und keiner universal gültigen Definition. Denn eine solche ist stark von der fachlichen Ausrichtung der jeweiligen Bildungsstätte abhängig und kann sich im Spannungsfeld von evidenzbasierten faktischen Auffassungen samt der Einhaltung wissenschaftlicher Gütekriterien wie Objektivität, Validität und Reliabilität bis hin zu hermeneutischen Zugängen, die dem Subjektivismus verhaftet sind, bewegen. Soll die Psychotherapie eine gänzlich akademische Disziplin werden, muss sie zunächst zwangsläufig mit den unterschiedlichen Wissenschaftsbegriffen von Universitäten korrespondieren und in weiterer Folge ihre eigene wissenschaftliche Haltung begründen. Dabei müssten sich die insgesamt 23 anerkannten Fachrichtungen der Psychotherapie insofern homogenisieren, als sie zu einem gemeinsam gültigen Wissenschaftsbegriff finden müssten. Werfen wir einen Blick auf die Psychoanalyse, sind sowohl Stimmen für eine wissenschaftliche Begründung der Disziplin wahrnehmbar als auch gegenteilige. Cremerius (1987, S. 1092) spricht sich dafür aus, die Psychoanalyse als Wissenschaft zu fördern und auch Segal (1986, S. 309) postuliert, dass Psychoanalytiker_innen per se Wissenschaftler_innen seien, da sie sich mit „psychische Realitäten“ auseinandersetzen würden. Doch wenn wir davon ausgehen, dass das Unbewusste den primären Forschungsgegenstand der Psychoanalyse darstellt (Warsitz & Küchenhoff 2015, S. 95), müssen wir annehmen, dass es sich bei der Realität, von der Segal spricht, um eine subjektive Realität handelt, die in einem Jenseits von messbaren Kausalbeziehungen, die das heutzutage vorherrschende reduktionistische Wissenschaftsverständnis prägen, liegt. Es ergibt sich also ein gewisses Vakuum „[z]wischen Geist und Wissenschaft“ (Adorno 2010 [1963], S. 183) oder um es mit den Worten Pazzinis (2022a, S. 159) wiederzugeben: „Wahrheit braucht Spiel, zumal sie ja nicht vorgegeben oder lediglich gut versteckt wäre.“ Wenn wir davon ausgehen, dass wir es mit einer beliebigen Universität zu tun haben, die sich der klassischen empirischen Wissenschaft verschrieben hat und einem zumindest latenten Leitbild folgt, das sich ein leises Credo à la „*Mach weiter! Fahre fort, immer mehr zu wissen!*“ (Lacan 1997 [1969/70], S. 104) auf die Fahnenstange geschrieben hat, könnte sie unter Umständen Gefahr laufen, ihren eigenen Gegenstand und in weiterer Folge ihre Identität zu verkennen. Die Psychoanalyse bringt und verlangt nach einer gewissen Freiheit (Diekhans und Pazzini 2021, S. 9). Sie kann, angelehnt an die Worte Adornos (2010 [1963], S. 181), als eine wissenschaftliche Disziplin, „eine geistige Gestalt dessen, was Goethe wie Hegel als Entäußerung forderten: Hingabe des Geistes an ein ihm Entgegenstehendes und Fremdes, in der er erst seine Freiheit gewinnt“ beschrieben werden. Die Psychotherapie als eine *Wissenschaft vom Subjektiven* (Pritz und Teufelhart 1996) und gleichzeitig Fremden. Ein Subjektives, dessen Begehren sich durch den anderen konstituiert? Eine Wissenschaft vom *anderen*? Baut doch die Psychotherapie unabhängig von der jeweiligen Schule auf Beziehung auf, nachdem der Mensch am *Nebenmenschen* erkennen lernt (Freud 1950c [1895], S. 426). Ein Nebenmensch, der uns bis zu einem gewissen Grad immer rätselhaft bleibt.

Bezugnehmend auf die zweite Frage, wie eine solche ausschließliche Akademisierung der Psychotherapie gelingen kann, ließen sich vielerlei verschiedene Aspekte diskutieren – beispielsweise politische, kulturanalytische oder ökonomische. Doch die vorliegende Diskussion möchte ein spezifisches Merkmal einer erfolgreichen und vollständigen universitären Etablierung der Psychotherapie aufgreifen: nämlich das Potenzial der Jugend. Wie bereits an anderer Stelle festgehalten wurde, kann davon ausgegangen werden, dass sich das Durchschnittsalter von Psychotherapie-Ausbildungskandidat_innen bzw. Studierenden im Zuge einer ausschließlichen Akademisierung des Fachs erheblich verringern würde. Während das Studium oder die Ausbildung heutzutage oftmals als Zweitstudium oder nach einer vorherigen Berufsausbildung absolviert wird, würde die Akademisierung zu einer vermehrten Aufnahme eines Erststudiums der Psychotherapie führen. Eine Entwicklung, die sich am Studium der Psychotherapiewissenschaft an der Sigmund Freud PrivatUniversität beobachten lässt. Innerhalb der Psychotherapie-Gemeinschaft bestehen teilweise erhebliche Zweifel gegenüber einem sinkenden Altersdurchschnitt der psychotherapeutischen Ausbildungskandidat_innen. Den Bedenken werden jedoch auch positive Aspekte eines niedrigeren Altersdurchschnitts wie beispielsweise eine hohe Motivation von Studienanfänger_innen (Rieken 2013, S. 296) und die oftmals günstigen Rahmenbedingungen hinsichtlich der Lebensrealitäten der Spätadoleszenten entgegengehalten (Fäh 2020, S. 567). Eine hinreichende Generationendiversität könnte darüber hinaus zu einer Bereicherung des gemeinsamen (Er‑)Lernens führen, nachdem Leerstellen der jeweiligen Generationen im gegenseitigen Austausch gegebenenfalls beleuchtet und befüllt werden können. Die Jugend ist unbestritten eine besondere Lebenszeit, in der eine Fülle an Entwicklungsschritten möglich ist. Auch Bohleber (1999, S. 526) schreibt dem Jugendalter eine besondere Dynamik zu und stellt eine Querverbindung zur Psychoanalyse her, wenn er schreibt: „Es ist ein Kennzeichen der Psychoanalyse, Natur und Kultur jeweils neu zu reflektieren und nicht das eine in dem anderen aufzulösen, sondern sich immer wieder auf die Zwischenposition, die sie einnimmt und die ihre Stärke ist, zu besinnen. Die Adoleszenz bildet eines der Felder, auf denen dies zum Tragen kommt.“ Die Psychotherapie könnte von diesem Potenzial der Jugend zur Reflexion und einem – salopp ausgedrückt – Offenhalten profitieren. Denn junge Studienbeginner_innen, die sich direkt im Anschluss an ihre schulische Ausbildung für ein Studium der Psychotherapie entscheiden, die sogenannten *Emerging Adults* (Arnett 2000), die sich in einem Übergang von Jugend und Erwachsenenalter befinden, nehmen die jugendliche Neugierde mit in ihr Studium, wodurch die gesamte Disziplin dynamisiert, und der wissenschaftliche Diskurs bereichert werden könnte. Freilich lassen sich eine gesteigerte Motivation und Interesse für die Sache nicht ausschließlich am Alter eines Menschen festmachen, sind doch innerhalb jeder Generation stets individuelle Unterschiede zu beobachten. Betrachten wir beispielsweise die aktuelle sogenannte *Generation Z*, sehen wir junge Menschen, die sich in sozialen Bewegungen wie *Fridays for Future* enthusiastisch engagieren. Sie blicken kritisch in die Welt, reflektieren ihre Positionen im Geschehen und haben durch den zunehmenden Verlust des Autoritären, des Väterlich-Symbolischen (Frühauf und Hartmann 2022, S. 9), wenig Berührungsängste, Gesetze infrage zu stellen und ihre Bedenken offen zu diskutieren sowie für ihre Werte, ihr Begehren, einzustehen. Nach Barthes (2010 [1972], S. 339) findet beim Forschen stets eine „Trennung der Diskurse“ statt. Einerseits gehe es um den Diskurs der Wissenschaftlichkeit, andererseits um den Diskurs des Begehrens. Wenn es den jungen Generationen also gelänge, diese Dichotomisierung der Diskurse zu überwinden und ihr Begehren mit einer Psychotherapiewissenschaft in Zusammenhang zu bringen? Läge dann darin nicht ein großes Potenzial für eine breite Begründung der Psychotherapie als wissenschaftliche Disziplin, die den gängigen Methodenkanon um alternative Wissenschaftsverständnisse (Rieken 2013, S. 295f.), die in den Hintergrund geraten sind, erweitert? Darüber hinaus bietet die Akademisierung der Psychotherapie als Schlüsselwissenschaft zwischen Geistes- und Naturwissenschaft die Möglichkeit einer ausgeprägteren Genderdiversität innerhalb der jeweiligen Disziplinen. Werden doch noch immer Studiengänge wie Philosophie, bei denen angenommen wird, dass eine gewisse Begabung vorhanden sein muss, vermehrt von Männern belegt und jene wie Psychologie, die mit einem Erlernen verbundenen werden, primär von Frauen gewählt (Leslie et al. 2015, S. 262). Eine Psychotherapiewissenschaft also, die mithilfe der „schöpferischen Funktion“ ihrer Studierenden (Benjamin 2010 [1915], S. 53) und ihrer Generationen- und Genderdiversität ein universitäres Fach begründet, das denen, die das Begehren haben, zugänglich ist und die Universität zu einem Ort „der Unabhängigkeit und Freiheit, der Einheit von Forschung und Lehre und der uneingeschränkten Ausrichtung der Wissenschaft auf Wahrheit“ (Unbedingte Universitäten 2010, S. 7) transformiert? Dann müsste sich Mensch nur noch auf die Wahrheit einigen.

## Ausblick

Auch wenn sich das Psychotherapiegesetz seit Jahrzehnten in der Praxis bewährt hat, wird es früher oder später zu einer Gesetzesnovellierung kommen, und zwar aus einem einfachen Grund: Weil das Fortschreiten der Zeit diese notwendig machen wird. Dabei sollte von Seiten politischer Akteur_innen als auch Psychotherapeut_innen beachtet werden, dass das Äußere das Innere und das Innere das Äußere maßgeblich beeinflussen wird. Egal an welchem Ort sich das, was „Ausbildung genannt wird und eher Bildung heißen könnte“ (Pazzini 2022b, S. 6), vollzieht. Nicht nur die Psychoanalyse unterliegt einem unendlichen Diskurs, der die Frage aufwirft, ob ihre Singularität nicht „dem entstammte, dass sie niemals beendet, niemals auf einen bereits abgeschlossenen Korpus reduzierbar wäre, auf ein bereits existierendes Wissen, auf ein bereits festgelegtes Gesetz, dass sie ‚nicht-endend‘ bliebe?“ (Irigaray 2022, S. 129), sondern auch die Psychotherapie im Allgemeinen. Diese Unabschließbarkeit der Bildung mag ein Grund dafür sein, weshalb Freud davon ausging, dass die Akademisierung des Fachs nicht zwangsläufig außeruniversitäre Institutionen abschaffe (Freud 1919j [1918], S. 700). Scheint doch der Gegenstand der Psychotherapie hinreichend groß zu sein, um ein sowohl als auch gewähren zu können. Es muss lediglich die „Konstruktion einer geschützten Raumzeit, darum, den Wunsch in ein Begehren und ein Sprechen umzuwandeln“ (Pazzini 2022b, S. 6) ermöglicht werden. Lediglich im Sinne einer anspruchsvollen Minimalanforderung, die voraussetzt, dass die Selektionsfunktion der Bildung aufgehoben wird – auch im Hinblick auf etwaige Altersbegrenzungen. Wenn wir nicht in die Jugend vertrauen – in wen dann? Somit könnten durch die Akademisierung der Psychotherapie nicht nur das Fach selbst und der vorherrschende wissenschaftliche Diskurs eine Bereicherung erfahren, sondern die Universität als Ganzes könnte ein Raum werden, an dem etwaige Generationsspannungen (King 2022, S. 1143) ab- und Generationsdiversität aufgebaut sowie Zusammenhänge zwischen Studienfach und Geschlechterverteilung ausgeglichen werden können. In diesem Sinne sollte sowohl der innere Eklektizismus der Psychotherapie in ihrer Methodenpluralität als auch die äußere Freiheit ihrer institutionellen Anbindung und Akademisierung erhalten und für einen gemeinsamen Diskurs offenbleiben. Denn bei all den Gesetzen und ihrer gleichzeitigen Unmöglichkeit müssen wir stets eine Ethik unserer Disziplin und ihre Auswirkung auf die klinische Praxis berücksichtigen. Darüber sollten wir sprechen.
